# Faintly tired: a systematic review of fatigue in patients with orthostatic syncope

**DOI:** 10.1007/s10286-022-00868-z

**Published:** 2022-06-10

**Authors:** Ryan E. Y. Wu, Farhaan M. Khan, Brooke C. D. Hockin, Trudie C. A. Lobban, Shubhayan Sanatani, Victoria E. Claydon

**Affiliations:** 1 Department of Biomedical Physiology and Kinesiology, Simon Fraser University, Burnaby, BC Canada; 2 Syncope Trust and Reflex Anoxic Seizures Group (STARS) and Arrhythmia Alliance, Stratford-upon-Avon, Warwickshire, UK; 3 Department of Pediatrics, University of British Columbia, Vancouver, BC Canada

**Keywords:** Syncope, Fatigue, Vasovagal, Postural orthostatic tachycardia syndrome, Orthostatic hypotension

## Abstract

**Background:**

Orthostatic syncope (transient loss of consciousness when standing—fainting) is common and negatively impacts quality of life. Many patients with syncope report experiencing fatigue, sometimes with “brain fog”, which may further impact their quality of life, but the incidence and severity of fatigue in patients with syncope remain unclear. In this systematic review, we report evidence on the associations between fatigue and conditions of orthostatic syncope.

**Methods:**

We performed a comprehensive literature search of four academic databases to identify articles that evaluated the association between orthostatic syncope [postural orthostatic tachycardia syndrome (POTS), vasovagal syncope (VVS), orthostatic hypotension (OH)] and fatigue. Studies were independently screened using a multi-stage approach by two researchers to maintain consistency and limit bias.

**Results:**

Our initial search identified 2797 articles, of which 13 met our inclusion criteria (POTS *n* = 10; VVS *n* = 1; OH *n* = 1; VVS and POTS *n* = 1). Fatigue scores were significantly higher in patients with orthostatic syncope than healthy controls, and were particularly severe in those with POTS. Fatigue associated with orthostatic syncope disorders spanned multiple domains, with each dimension contributing equally to increased fatigue. “Brain fog” was an important symptom of POTS, negatively affecting productivity and cognition. Finally, fatigue was negatively associated with mental health in patients with POTS.

**Conclusion:**

In conditions of orthostatic syncope, fatigue is prevalent and debilitating, especially in patients with POTS. The consideration of fatigue in patients with orthostatic disorders is essential to improve diagnosis and management of symptoms, thus improving quality of life for affected individuals.

**Supplementary Information:**

The online version contains supplementary material available at 10.1007/s10286-022-00868-z.

## Introduction

Orthostatic syncope (fainting, transient loss of consciousness and postural tone when upright) and presyncope (near-fainting) are common and primarily occur due to a reduction in cerebral blood flow when upright, with spontaneous recovery of symptoms upon removal of the orthostatic stress [1]. Orthostatic syncope is prevalent, with an incidence within the general population of between 5.7 and 6.2 per 1000 person-years [2]. The typical age of first episode of symptoms is between 10 and 15 years [3–5], with 15–25% of children and adolescents experiencing at least one episode of syncope before adulthood [6]. A second peak of symptoms occurs in later life (~ 70 years of age), with incidence rates increasing to 11.1 per 1000 person-years in older adults [3]. Orthostatic syncope is typically considered to be a benign condition because it is not immediately life-threatening, but in both younger and older patients it has a profound negative impact on quality of life and the ability to participate in activities of daily living [7, 8]. Syncope is also associated with significant injuries, resulting in increased hospitalizations, and costing patients US $1.7 billion and CAD $50 million annually within the USA and Canada, respectively [9–11].

While all orthostatic syncopal and presyncopal disorders are associated with at least transient autonomic dysfunction and share common features, there are several distinct subtypes of orthostatic syncope. Vasovagal syncope (VVS) is the most commonly encountered form of orthostatic syncope, and is typically observed in children and young adults [2, 12]. VVS is a form of reflex syncope, and is associated with sudden onset hypotension and bradycardia accompanied by symptoms of cerebral hypoperfusion that culminates in loss of consciousness [13]. In very young children, a dominant cardioinhibitory form of VVS, known as reflex asystolic syncope (RAS), can present, in which the bradycardic component is particularly pronounced [14].

Postural orthostatic tachycardia syndrome (POTS) is also a common cause of orthostatic intolerance, affecting 0.5–3 million individuals within the USA, and is associated with frequent and debilitating orthostatic presyncopal symptoms [15]. POTS occurs due to abnormal haemodynamic compensation when standing [16] and is associated with excessive orthostatic heart rate increases that compromise cardiac output, with consequent cerebral hypoperfusion and presyncopal symptoms [17]. In the absence of significant hypotension, loss of consciousness is not common in patients with POTS.

In older adults, OH is the most common form of syncope, with a prevalence of 30% in those greater than 70 years of age [18]. OH occurs when the typical reflex adaptations to orthostatic stress fail, due to structural or functional impairments to autonomic responses that mediate the restoration of blood pressure when upright [17, 19]. Accordingly, in patients with OH, blood pressure typically progressively declines with orthostatic stress, with a failure to increase heart rate [20]. When blood pressure is no longer sufficient to support adequate cerebral perfusion, symptoms of presyncope and syncope occur [21, 22]. Elderly individuals are also reported to experience syncope secondary to carotid sinus hypersensitivity (CSH), but in this case the primary trigger is thought to be mechanical stimulation of the carotid baroreceptors, e.g. due to neck turning, that provokes paradoxical bradycardia and hypotension [23]. While CSH is exacerbated by orthostatic stress, it is not strictly an orthostatic syncopal disorder.

One common symptom associated with all forms of orthostatic syncope is chronic fatigue, which is defined as an immense sensation of both physical and mental tiredness or exhaustion [24] that is not relieved by rest or sleep [25]. Fatigue in patients with syncope is a concern as it has a debilitating effect on activity levels, quality of life and the efficacy of pharmacological treatments [26, 27]. In addition, fatigue is associated with depression, enhanced irritability, reduced productivity, increased stress and decreased memory function [28]. Fatigue also negatively affects sleep quality, with reports that up to 32% of adults with POTS have sleep disturbances due to fatigue [29, 30]. The common association between fatigue and all forms of orthostatic syncope may reflect a common mechanism, through cerebral hypoperfusion, which is regularly noted in patients with orthostatic syncope when in an upright position [31]. However, it may be that fatigue is associated more with some syncopal subtypes than others. For example, fatigue and a related sensation of “brain fog” (described as a lack of mental clarity) are reported to be particularly notable in patients with POTS [32]. It is imperative to understand the associations between the different syncope subtypes and fatigue to better enable recognition and treatment of fatigue in order to improve quality of life for those living with syncopal disorders. Accordingly, we aimed to perform a systematic review of the available literature to obtain a deeper understanding of the prevalence, severity, predisposing factors and consequences of fatigue in patients with orthostatic syncope. In addition, we performed a meta-analysis of available data, where possible, to determine the effects of syncope subtypes on fatigue severity.

## Methods

### Search strategy

The databases used to search for published and peer-reviewed studies in all languages were as follows: MEDLINE (PubMed), Web of Science, PsychINFO and Cumulative Index to Nursing and Allied Health Literature (CINAHL), with all searches being conducted on 12 May 2021. The search approach combined clinical terms for orthostatic syncopal disorders with terms related to fatigue (Supplementary Table 1).

### Study selection and eligibility

Records obtained were uploaded to the reference management software Zotero (version 5.0.96.2). Duplicate items were then removed, and the remaining records were screened using a multi-stage approach. Articles were first screened using their title to exclude studies that were clearly unrelated to the research question, erring on the side of inclusion to ensure that no potentially relevant papers were missed. Articles that were not published in English were translated if title screening indicated that the study may be relevant to the research question. This is an important equity consideration as it prevents deprioritization of research reported in languages other than English.

Selected titles were then filtered based on abstracts to determine those that would be chosen for full-text screening. At this stage, screening was focussed on ensuring that included articles would be relevant to the research question. Studies were only selected for full-text screening if they were published in a peer-reviewed journal. The chosen full-text articles were then screened with an emphasis on their viability for data extraction. The primary population in the included full-text articles was adults or children with a physician diagnosis (included self-reported physician diagnosis) of orthostatic syncope or presyncope in whom an assessment of fatigue had been conducted. The population must have had at least one episode of syncope within the preceding year to be included. Those with syncope were required to have an autonomic aetiology, and accordingly patients with VVS, POTS, OH (sometimes referred to as autonomic failure) and carotid sinus hypersensitivity (CSH) were included. Lastly, studies were also required to utilize an established fatigue instrument as an outcome measure.

Articles were excluded during study selection if one of the following applied: the article was not published in a peer-reviewed journal; the article was a case report, case series (identified as five cases or fewer) or literature review; the article did not include terms related to syncope or fatigue; the patient population considered syncope as a consequence of autonomic dysfunction related to corona virus disease 2019 (COVID-19), or patients with syncope that was not orthostatic in nature (such as syncope secondary to arrhythmia, structural heart disease, metabolic disease or epilepsy); the article referred to chronic fatigue syndrome without the mention of orthostatic syncope or associated conditions. Articles for which the full text was not available, or for which established fatigue instruments were not used or data were not reported were also excluded.

All articles were independently screened by two researchers (R.E.Y.W. and F.M.K.) to avoid bias. Any contentions were ameliorated with the consultation of a third researcher who was an expert in the subject area (V.E.C.).

### Patient-oriented research perspective

We worked with Syncope Trust and Reflex Anoxic Seizures (STARS), a community partner and patient advocacy group, to evaluate whether the objective numerical data identified through the literature evaluation resonated with the subjective lived experience of patients with orthostatic syncope. This integrated knowledge translation approach strengthens the applicability of the research. Our community partners were not involved in the literature search, article selection process or data extraction and analysis, but reviewed the study results and provided their perspectives on the knowledge summaries based on the lived experience of the patient community and consideration of how the results compared with community surveys.

### Data extraction

Data were extracted from full-text articles into a single spreadsheet as follows: publication date, lead author, the country of research, study design, sample size, participant age, sex and race, the duration and frequency of participant symptoms, the primary orthostatic syncope disorder(s) evaluated, the presence of a control group, the demographics of the control or comparison group (if present), the fatigue instrument(s) employed, the statistical comparison(s) made, the primary results, any additional noteworthy results and relevant statistical approaches/results, any explanatory relationships identified, and comparisons made with other populations. All data were independently extracted by two researchers (R.E.Y.W. and F.M.K.) to alleviate potential biases.

For studies where data were presented only in figures, means and standard deviations were estimated and extracted using WebPlotDigitizer (version 4.4; Pacifica, CA, USA), a publicly available tool.

Data that were presented as median ± range or interquartile range or mean ± standard error were calculated and converted to mean ± standard deviation using RStudio (version 1.4.1717) using a standard approach [33].

### Statistical analysis

This literature review aimed to compile all available fatigue scores from syncope patients and identify the prevalence and severity of this symptom. Where possible, comparisons between fatigue scores in patients with orthostatic syncope and healthy controls were performed (unpaired *t* test). Where the same fatigue instruments were used in multiple studies, pooled means and standard deviations were calculated. Then, using a one-way analysis of variance (ANOVA), the weighted means and standard deviations were evaluated against population reference data and comparisons of fatigue across unique patient populations were performed to identify the potential effects of syncope subtypes on fatigue. When only two sets of data were available, a paired *t* test was performed. All calculations were performed using SigmaPlot (version 14.0), and results were determined to be statistically significant where *p* < 0.05.

## Results

### Study characteristics

**Fig. 1:**
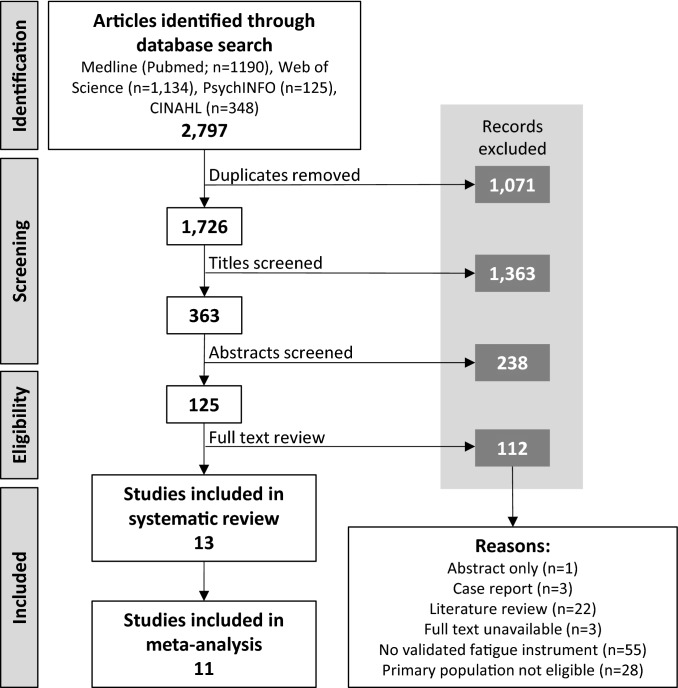
Preferred Reporting Items for Systematic reviews and Meta-Analyses (PRISMA) diagram showing the process for identifying eligible articles for inclusion within the review. Adapted from PRISMA statement [90]

The initial search of the databases yielded 2797 articles; following a rigorous screening process, 13 articles that met all the inclusion criteria were included in this review (Fig. 1).

Table 1 describes the study characteristics for the 13 identified articles organized by the primary population investigated for fatigue. The primary populations within the included articles were as follows: ten POTS (*n* = 1226), one VVS (*n* = 91), one OH (*n* = 40), and one study examined both patients with POTS and VVS (*n* = 249). No studies that investigated fatigue in patients with CSH or RAS were identified. Most studies were cross-sectional in design (61%) [34–41], with a further 39% incorporating a case–control design [42–46]. Only three of the included articles reported data on participant race [34, 35, 40], and only six studies presented the duration of syncope symptoms within their sample [34–36, 42–45]. Three studies provided the frequency of recurrent syncope within the population of interest [34, 35, 41]. All articles identified represented the North American experience, with the exception of two studies examining fatigue in patients with POTS living in the UK.

**Table 1:** Study characteristics

Study	Country	Study design	Sample size (% female)	Age (years)	Race	Duration of syncope diagnosis (years)	Frequency of recurrent syncope		Fatigue measure(s) employed	
*Postural orthostatic tachycardia syndrome (POTS)*										
Wise et al. [34]	USA	Cross-sectional	133 (90)	20 ± 5	99% White 4% Hispanic 1% Other	5.8 ± 4.6	29% yearly 8% monthly 12% weekly 1% daily		MFTQ WMFI	
Ross et al. [35]	USA	Cross-sectional	138 (88)	20 ± 5	99% White 4% Hispanic 1% Other	5.8 ± 4.6	29% yearly 8% monthly 12% weekly 1% daily		WMFI	
Baker et al. [36]	Canada	Cross-sectional	30 (53)	27 ± 4	–	1	–		MFI	
Pederson et al. [42]	USA	Case–control	624 (97)	34 ± 11	–	10	–		FSS	
Miglis et al. [43]	USA	Case–control	18 (72)	27 ± 12	–	≥ 0.5	–		FSS	
Rea et al. [44]	USA	Case–control	32 (88)	26 ± 10*	–	–	–		FSS	
McDonald et al. [37]	UK	Cross-sectional	136 (90)	33 ± 10	–	–	–		FIS	
Lewis et al. [38]	UK	Cross-sectional	24 (79)	29 ± 12	–	–	–		FIS Chalder fatigue scale	
Okamoto et al. [39]	USA	Cross-sectional	47 (100)	30 ± 14	–	–	–		CIS RAND-36	
Bagai et al. [45]	USA	Case–control	44 (89)	36 ± 11	–	≥ 0.5	–		RAND-36 F-VAS	
*Vasovagal syncope (VVS)*										
Legge et al. [46]	USA	Case–control	91 (78)	55 ± 21	–	–	–		FIS	
*Orthostatic hypotension (OH)*										
Wecht et al. [40]	USA	Cross-sectional	40 (48)	63 ± 11	48% Black	–	–		OHSA	
*Vasovagal syncope (VVS) and postural orthostatic tachycardia syndrome (POTS)*										
Hall et al. [41]	USA	Cross-sectional	POTS: 177 (93) VVS: 72 (67)	POTS: 31 ± 11* VVS: 34 ± 14*	–	–		POTS: ≥ 1 × in lifetime VVS: ≥ 3 × in lifetime		RAND-36

Data presented as mean ± standard deviation or percentage as appropriate

*Standard deviation estimated from range or standard error of mean

Note that Wise et al. (2015) and Ross et al. (2013) reported data collected from the same patient sample

*CIS* checklist of individual strength; *FIS* fatigue impact scale; *FSS* fatigue severity scale; *F-VAS* fatigue visual analogue scale; *MFI* multidimensional fatigue inventory; *MFTQ* myalgic encephalomyelitis/chronic fatigue syndrome fatigue type questionnaire; *OHSA* orthostatic hypotension symptoms assessment; *POTS* postural orthostatic tachycardia syndrome; *RAND-36* RAND-36 item health survey; *VVS* vasovagal syncope; *WMFI* Wood mental fatigue inventory

In total, ten distinct fatigue instruments were used: the fatigue impact scale (FIS) (*n* = 3), the fatigue severity scale (FSS) (*n* = 3), the RAND 36-item health survey (RAND-36) (*n* = 3), the Wood mental fatigue inventory (WMFI) (*n* = 2), the Chalder fatigue scale (*n* = 1), the checklist of individual strength (CIS) (*n* = 1), the fatigue visual analogue scale (FVAS) (*n* = 1), the orthostatic hypotension symptoms assessment (OHSA) (*n* = 1), the myalgic encephalomyelitis/chronic fatigue syndrome fatigue type questionnaire (MFTQ) (*n* = 1), and the multidimensional fatigue inventory (MFI) (*n* = 1) (Table 1). Details of the fatigue instruments used can be found in Supplementary Table 2.

### Evidence on the association between fatigue and orthostatic syncope

Table 2 contains all study results and is arranged according to the primary patient population. In seven studies, comparisons were made to a control group or reference population. Of these studies, one evaluated fatigue with the MFI [36], three with the FSS [42–44], one with the RAND-36 [45] and the F-VAS [45], one with the FIS [46], and one using the OHSA [40]. In six of the seven studies, patients with orthostatic syncope had significantly more fatigue than healthy controls, regardless of the syncope subtype (POTS [42–45], VVS [46], or OH [40]), or the fatigue instrument employed [40, 42–46]. However, one study examining patients with POTS using the MFI had conflicting results, noting that fatigue was less severe than in a comparison group of healthy controls [36]. One study qualified the incidence of “severe” fatigue (based on a CIS score > 36) and found it to be present in 93% of patients with POTS [39].

**Table 2:** Study results

Study	Outcome measure (s)	Fatigue severity	Comparisons	Noteworthy additional results
*Postural orthostatic tachycardia syndrome (POTS)*				
Wise et al. [34]	MFTQ & WMFI	MFTQ scores: Post-exertional fatigue: 184 ± 67^‡^ Wired/pain fatigue: 121 ± 80^‡^ Brain fog fatigue: 163 ± 67^‡^ WMFI scores: 23.9 ± 8.7	Factor analyses revealed that patients with POTS experience fatigue as a multidimensional construct with three dimensions: post-exertional fatigue, wired/pain fatigue and brain fog fatigue	Two patient groups were created based on the severity of their fatigue symptoms. Those in the high fatigue severity group (*n* = 55) had higher scores than those with low fatigue (*n* = 78) in all subdomains of fatigue: post-exertional (280 ± 65 versus 116 ± 69), mentally wired/pain (179 ± 98 versus 79 ± 67) and brain fog (241 ± 77 versus 108 ± 60)
Ross et al. [35]	WMFI	WMFI scores: 23.9 ± 8.7	Brain fog severity ratings were significantly correlated with WMFI scores, indicating that brain fog is a major component of fatigue (*r* = 0.512, *p* < 0.0001)	Of all the patients with POTS (*n* = 138), 96% experienced brain fog at least once (*n* = 132) and 67% experienced brain fog on a daily basis (*n* = 93). Brain fog was reported to impair the ability to participate in social activities (92%), work (66%) and school (96%) The most frequent triggers of brain fog were physical fatigue (91%), a lack of sleep (90%), prolonged standing (87%), dehydration (86%) and faintness (85%) Those with sleep disorders (*n* = 102) had significantly higher scores on the WMFI in comparison with those without sleep disorders (*n* = 36) (24.9 ± 6.7 versus 21.9 ± 9.5, *p* < 0.01)
Baker et al. [36]	MFI	MFI scores: Total: 8.83 ± 3.09^†^ General fatigue: 8.81 ± 6.12^‡^ Physical fatigue: 6.37 ± 4.01^‡^ Reduced activity: 6.95 ± 4.56^‡^ Reduced motivation: 6.90 ± 4.25^‡^ Mental fatigue: 9.65 ± 7.17^‡^	Fatigue scores were lower in the POTS population (*n* = 30) than a healthy control reference population (*n* = 222) [84] in terms of general fatigue (8.81 ± 6.12^‡^ versus 12.9 ± 4.7), physical fatigue (6.37 ± 4.01^‡^ versus 10.9 ± 4.4), reduced activity (6.95 ± 4.56^‡^ versus 9.3 ± 4.2), reduced motivation (6.90 ± 4.25^‡^ versus 9.6 ± 3.9) and mental fatigue (9.65 ± 7.17^‡^ versus 10.9 ± 4.5). Subdomain scores were not significantly different between each other, indicating they all contribute equally to fatigue in patients with POTS	Changes in patient heart rate to head up tilt during 1-year follow-up did not significantly correlate with general fatigue (*r* = 0.006; *p* = 0.97). Severity of orthostatic symptoms was associated with fatigue scores at 1-year follow-up (*r* = 0.374, *p* < 0.05)
Pederson et al. [42]	FSS	FSS scores: 56.2 ± 8.7	Patients with POTS (*n* = 624) reported significantly higher levels of daytime fatigue than healthy controls (*n* = 139) (56.2 ± 8.7 versus 31.2 ± 13.6, *p* < 0.001). Patients with POTS had fewer days with good energy than controls (2.26 ± 4.07 versus 12.34 ± 9.57, *p* < 0.001) and more days with brain fog than controls (19.34 ± 9.97 versus 5.19 ± 8.99, *p* < 0.001)	The POTS group (*n* = 624) reported poorer sleep quality (based on the Pittsburgh Sleep Quality Index) compared with the controls (*n* = 139), as indicated by their higher scores (13.3 ± 4.1 versus 7.5 ± 4.0, *p* < 0.001) Compared with controls, patients with POTS, who had more fatigue, had a higher suicide risk (*p* < 0.001), were more likely to have formed at least one suicide plan (*p* = 0.01) and were more likely to have threatened to make a suicide attempt or attempted suicide in the past (*p* < 0.001). Lastly, when asked about the likelihood of attempting suicide in the future, significantly more patients with POTS reported that they were likely to do so when compared with healthy controls (*p* = 0.01)
Miglis et al. [43]	FSS	FSS scores: 50.9 ± 11.5*	Those with POTS (*n* = 18) reported significantly higher levels of fatigue when compared with controls (*n* = 16) (50.9 ± 11.5 versus 40.7 ± 12.9, *p* = 0.004)*	There was no difference in subjective sleepiness between patients with POTS (*n* = 18) and healthy controls (*n* = 16) when considering the responses to the Epworth sleepiness scale (8.1 ± 5.1 versus 7.5 ± 7.4, *p* = 0.822)*
Rea et al. [44]	FSS	FSS scores: 54.0 ± 13.5*⁑	FSS scores were significantly higher in patients with POTS (*n* = 32) than healthy controls (*n* = 32) (54.0 ± 13.5 versus 26.1 ± 7.2, *p* < 0.0001*)	FSS scores uniquely contributed to severity of autonomic dysfunction measured using the COMPASS-31
McDonald et al. [37]	FIS	FIS Scores: 92 ± 34	–	There were no differences between FIS scores in POTS patients recruited through a POTS patient support group (*n* = 84) and those recruited through a falls and syncope clinic (*n* = 52) (91.0 ± 32.0 versus 92.0 ± 37.0, *p* = 0.8)
Lewis et al. [38]	FIS & Chalder fatigue scale	FIS scores: 101 ± 34 Chalder fatigue scale: Total: 8 ± 4 Physical fatigue: 5 ± 3 Mental fatigue: 3 ± 2 (Note Chalder fatigue scores provided as percentage maximum rather than according to scoring convention)	–	All POTS patients in this study also met criteria for CFS (*n* = 24). When these patients with CFS and POTS were compared with a cohort with CFS without POTS (*n* = 155), there were no significant differences in FIS scores between those with CFS-POTS and those with CFS without POTS (101.0 ± 34.0 versus 98.0 ± 34.0, *p* = 0.7). Chalder fatigue scale scores were lower in those with CFS-POTS than those with CFS without POTS indicating less fatigue in terms of total fatigue (8.0 ± 4.0 versus 10.0 ± 2.0, *p* < 0.01) as well as domains of physical fatigue (5.0 ± 3.0 versus 6.0 ± 1.6, *p* < 0.01) and mental fatigue (3.0 ± 2.0 versus 3.0 ± 1.0, *p* < 0.01). CFS-POTS patients had greater orthostatic intolerance (*p* < 0.0001) and more severe autonomic dysfunction than those with CFS without POTS
Okamato et al. [39]	CIS & RAND-36	CIS scores: fatigue subscale 48.1 ± 8.6*† RAND-36 Energy and Fatigue Score: 22.1 ± 19.6*†	Two patient groups were created based on the presence of diagnostic criteria for CFS. Those within the CFS-POTS group (*n* = 30) reported fatigue that was statistically more severe than those in the non-CFS-POTS group (*n* = 17), when considering CIS scores (51 ± 5.5 versus 43 ± 12.4, *p* = 0.016). The CFS-POTS group had significantly lower scores on the energy and fatigue subdomain of the RAND-36 when compared with the non-CFS-POTS group (16.8 ± 16.4 versus 31.5 ± 25.2, *p* = 0.037)	Both patients with CFS-POTS (*n* = 30) and non-CFS-POTS (*n* = 17) reported extremely low scores in the role limitation subdomain of the RAND-36 (0.9 ± 4.9 versus 2.9 ± 11.9, *p* = 0.676). Physical functioning scores of the RAND-36 were also lower in the CFS-POTS group than the non-CFS-POTS group, although the difference was not significant (32.8 ± 17.0 versus 40.1 ± 16.9, *p* = 0.153). Moreover, severe fatigue was observed in 93% (CIS > 36) of patients with POTS
Bagai et al. [45]	F-VAS & RAND-36	RAND-36 Energy and Fatigue Score: 30.0 ± 7.0 F-VAS Scores: 7.5 ± 2.0	Patients with POTS (*n* = 44) reported significantly greater levels of fatigue than healthy controls (*n* = 46) (7.5 ± 2.0 versus 2.8 ± 2.5, *p* < 0.0001). Patients with POTS also had significantly lower scores in the energy and fatigue subdomain of RAND-36 in comparison with controls (30.0 ± 7.0 versus 54.0 ± 10.0, *p* < 0.0001)	The Medical Outcomes Study Sleep Problems Index identified significantly more sleep problems in patients with POTS (*n* = 44) than the control group (*n* = 46) as indicated by the higher scores (58.0 ± 18.0 versus 20.0 ± 13.0, *p* < 0.0001) and strong correlation between sleep problems and F-VAS (*r* = 0.72, *p* < 0.0001) The Epworth sleepiness scale also found significantly higher levels of sleep disturbances in patients with POTS than controls as indicated by the higher scores (10.2 ± 5.7 versus 6.2 ± 3.7, *p* = 0.001)
*Vasovagal syncope (VVS)*				
Legge et al. [46]	FIS	FIS Scores: 26.0 ± 32.0	Patients with VVS (*n* = 91) were significantly more fatigued than healthy controls (*n* = 91) (26.0 ± 32.0 versus 13.0 ± 14.0, *p* < 0.0004)	Patients with VVS (*n* = 91) had significantly higher levels of daytime sleepiness than healthy controls (*n* = 91) as indicated by higher scores on the Epworth sleepiness scale (ESS) (7.0 ± 4.0 versus 3.0 ± 4.0, *p* < 0.0001). FIS scores were significantly correlated with ESS (*p* < 0.0001), and severity of autonomic dysfunction measured using the COMPASS-72 assessment (*p* < 0.0001)
*Orthostatic hypotension (OH)*				
Wecht et al. [40]	OHSA	OHSA Fatigue Subdomain Score: 3.5 ± 4.0*‡	Individuals with OH (*n* = 40) reported significantly more severe fatigue than reference scores (*n* = 184) (3.5 ± 4.0 versus 2.2 ± 2.6, *p* = 0.009*‡)	Individuals with delayed OH had more fatigue than those with OH occurring in the first 3 min of standing. Fatigue was more severe in older individuals with OH
*Vasovagal syncope (VVS) & postural orthostatic tachycardia syndrome (POTS)*				
Hall et al. [41]	RAND-36	POTS RAND-36 Energy and Fatigue Score: 27.2 ± 17.3* VVS RAND-36 Energy and Fatigue Score: 50.7 ± 22.1*	Two patient groups were created based on the diagnosis of the orthostatic syncope subtype Scores from the energy and fatigue subdomain of the RAND-36 were significantly lower in patients with POTS (*n* = 177) in comparison with patients with VVS (*n* = 72) (*p* < 0.001)	Male patients with POTS (*n* = 12) scored significantly lower than their female counterparts (*n* = 165) in the energy and fatigue subdomain of the RAND-36 (17.1 ± 14.5 versus. 27.9 ± 18.0, *p* = 0.024*). There were no differences in fatigue subdomain scores in patients with VVS between males (*n* = 24) and females (*n* = 48) (56.3 ± 23.0 versus 47.9 ± 21.5, *p* = 0.147*)

Data presented as mean ± standard deviation, unless otherwise stated

Note that Wise et al. (2015) and Ross et al. (2013) reported data collected from the same patient sample

*CFS* chronic fatigue syndrome; *CIS* checklist of individual strength; *COMPASS* composite autonomic symptom scale; *FIS* fatigue impact scale; *FSS* fatigue severity scale; *F-VAS* fatigue visual analogue scale; *MFI* multidimensional fatigue inventory; *MFTQ* myalgic encephalomyelitis/chronic fatigue syndrome fatigue type questionnaire; *OH* orthostatic hypotension; *OHSA* orthostatic hypotension symptoms assessment; *POTS* postural orthostatic tachycardia syndrome; *RAND-36* RAND-36 item health survey; *VVS* vasovagal syncope; *WMFI* Wood mental fatigue inventory

*Mean or standard deviation estimated from sample size, median and interquartile range, standard error of mean, or confidence intervals

^†^Data derived from the weighted average of all participants

^‡^Data extracted from figures

^⁑^Data approximated through scale conversion

One study compared patients with POTS to those with VVS, and found fatigue to be significantly greater in those with POTS than with VVS when evaluated using the energy and fatigue subdomain of the RAND-36, indicating more severe fatigue and lower levels of energy in those with POTS than with VVS [41].

In terms of types of fatigue experienced, several studies noted that the presence of brain fog was prevalent in patients with POTS [34, 35, 42]. Ross et al. noted that, in patients with POTS, 96% had experienced brain fog and 67% reported experiencing it daily, with a strong association between fatigue and brain fog [35]. This was also noted by Pederson et al., who observed that patients with POTS experienced more days with brain fog than controls [42]. Using the MFI, Baker et al. showed that fatigue in patients with POTS spanned multiple domains, with similar scores in each domain (general fatigue, physical fatigue, activity, motivation, and mental fatigue) suggesting that they all contribute equally to fatigue in POTS [36]. The notion that fatigue was multidimensional in patients with POTS was also noted by Wise et al., who found using the MFTQ that patients with POTS experienced fatigue as post-exertional fatigue, wired/pain fatigue, and brain fog fatigue [34]. In addition, compared with controls, patients with POTS were noted to have greater daytime fatigue and fewer days with good energy [42]. The two studies that evaluated fatigue in patients with VVS or OH did not report data on domains of fatigue or brain fog [40, 41].

### Factors influencing fatigue in patients with orthostatic syncope

Two studies examined the relationship between the severity of autonomic dysfunction and fatigue, in patients with POTS [44] and with VVS [46]. In both cases, more severe autonomic dysfunction as inferred using the COMPASS Composite Autonomic Symptom Scale was associated with more severe fatigue. However, interestingly, Baker et al. noted that severity of POTS, based on the magnitude of the orthostatic heart rate rise, was not correlated with fatigue, but rather that fatigue severity was correlated with the orthostatic symptom severity [36].

Two studies examined the severity of fatigue in patients with POTS who also met diagnostic criteria for CFS [38, 39]. Okamoto et al. found that fatigue measured using the CIS was significantly more severe in POTS patients meeting CFS criteria than in POTS patients who did not also meet criteria for CFS [39]. The CFS-POTS groups also had significantly lower scores on the energy and fatigue subdomain of the RAND-36 when compared with the patients with POTS who did not meet CFS criteria [39]. Lewis et al. found no significant differences in fatigue when comparing FIS scores between patients with CFS-POTS and patients with CFS who did not meet criteria for POTS (*p* = 0.7), and in fact those within their CFS-POTS cohort had lower total fatigue scores on the Chalder fatigue scale than the patients with CFS who did not meet diagnostic criteria for POTS (*p* < 0.001) (but greater orthostatic intolerance and autonomic dysfunction) (Table 3) [38].

**Table 3:** Comparison of study fatigue scores with reference data

Outcome measure	POTS	VVS	OH	References
*MFTQ*				
Wise et al. [34]				
Post-exertional fatigue	184 ± 67*			10.69 ± 25.44 [85]
Wired/pain fatigue	121 ± 80*			32.7 ± 50.2 [85]
Brain fog fatigue	163 ± 67*			25.8 ± 48.4 [85]
*WMFI*				
Ross et al. [35]	23.9 ± 8.7*			7.7 ± 5.1 [86]
Wise et al. [34]	23.9 ± 8.7*			
*MFI*				
Baker et al. [36]				
General fatigue	8.81 ± 6.12*			12.9 ± 4.7 [84]
Physical fatigue	6.37 ± 4.01*			10.9 ± 4.4 [84]
Reduced activity	6.95 ± 4.56*			9.3 ± 4.2 [84]
Reduced motivation	6.90 ± 4.25*			9.6 ± 3.9 [84]
Mental fatigue	9.65 ± 7.17*			10.9 ± 4.5 [84]
*FSS*				
Pederson et al. [42]	56.2 ± 8.7*			31.2 ± 13.6 [87]
Miglis et al. [43]	50.9 ± 11.5*			40.7 ± 12.9 [87]
Rea et al. [44]	54.0 ± 13.5*			26.1 ± 7.2 [87]
*FIS*				
McDonald et al. [37]	92 ± 34*			13.0 ± 14.0 [83]
Lewis et al. [38]	101 ± 34*			
Legge et al. [46]		26.0 ± 32.0*		
*CIS fatigue*				
Okamoto et al. [39]	48.1 ± 8.6*			23.0 ± 10.8 [88]
*RAND-36 energy and fatigue*				
Okamoto et al. [39]	22.1 ± 19.6*			52.2 ± 22.4 [89]
Bagai et al. [45]	30.0 ± 7.0*			
Hall et al. [41]	27.2 ± 17.3*	50.7 ± 22.1		
*F-VAS*				
Bagai et al. [45]	7.5 ± 2.0*			2.8 ± 2.5 [82]
OHSA fatigue				
Wecht et al. [40]			3.5 ± 4.0*	2.2 ± 2.6 [40]

Data presented as mean ± standard deviation. Patient data are as reported in Table 2, and reference data are as provided in the manuscript where comparisons with a control group were made in the original article, or as stated in Supplementary Table 2 (numbers in parentheses denote citations for reference data)

Note that data obtained using the Chalder fatigue scale (Lewis et al. 2013) could not be compared with reference data because the scores were provided as percentage maximum rather than according to scoring convention. Note that Wise et al. (2015) and Ross et al. (2013) reported data collected from the same patient sample

*CIS* checklist of individual strength; *COMPASS* composite autonomic symptom scale; *FIS* fatigue impact scale; *FSS* fatigue severity scale; *F-VAS* fatigue visual analogue scale; *MFI* multidimensional fatigue inventory; *MFTQ* myalgic encephalomyelitis/chronic fatigue syndrome fatigue type questionnaire; *OH* orthostatic hypotension; *OHSA* orthostatic hypotension symptoms assessment; *POTS* postural orthostatic tachycardia syndrome; *RAND-36* RAND-36 energy and fatigue score; *VVS* vasovagal syncope; *WMFI* Wood mental fatigue inventory.

*Significant difference from reference data

Several studies (*n* = 4) investigated the relationships between fatigue in patients with orthostatic syncope and sleep disturbances. Four papers reported that patients with POTS [35, 42, 45] and VVS [46] have significant sleep problems, including poorer sleep quality and greater levels of sleep disturbances, relative to healthy controls [35, 42, 45, 46]. Several studies identified that those with sleep problems had more severe fatigue [35, 45, 46]. Interestingly, two studies considered whether patients with POTS had excessive daytime sleepiness using the Epworth sleepiness scale, with conflicting results [43, 45]. One study found that patients with VVS had higher scores on the Epworth sleepiness scale than healthy controls, indicating increased daytime sleepiness, and noted that the sleepiness scores were significantly correlated with the fatigue severity [46].

Potential relationships between fatigue and mental health were also explored. Suicide risk, suicide attempts and the likelihood of attempting suicide in the future were significantly higher than healthy controls in patients with POTS, who had higher levels of fatigue than healthy controls [42].

Brain fog is a cognitive impairment resulting in difficulty with focussing and is sometimes conflated with fatigue. As noted above, symptoms of brain fog were commonly described in patients with POTS [34, 35, 42] (Table 3). The presence of brain fog was associated with a negative impact on the ability to participate in social activities,work, and school [35]. The factors most likely to trigger brain fog symptoms were physical fatigue, lack of sleep, prolonged standing, dehydration and faintness [35].

One study investigated the relationship between sex, syncope and fatigue [41]. An evaluation of male and female patients with POTS revealed that fatigue severity was influenced by sex, whereby male patients with POTS had significantly lower scores in the energy and fatigue subdomain of the RAND-36 than females, signifying more fatigue [41]. However, in the same study, there were no sex differences in fatigue severity in patients with VVS [41].

One study examined the impact of age on fatigue in patients with OH, using the OHSA fatigue subdomain score, and identified more severe fatigue in older individuals with OH, and particularly in those with the delayed form of OH [40].

### Meta-analysis of syncope subtypes and fatigue

We compared fatigue scores in the identified studies with reference values in healthy control cohorts (Table 3). These reference values were either those provided for a control group included in the original study, or where these data were not provided, population reference/normative data were used for comparison. Using this approach, fatigue scores in the 14 comparisons for patients with POTS were significantly more severe than the reference population in every case, with the exception of the data obtained using the MFI by Baker et al. (Table 3). Similar comparisons were possible for two studies examining patients with VVS, one of which identified significantly worse fatigue than in healthy controls [46] and one of which found no differences between the patients with VVS and healthy controls [41].

**Fig. 2:**
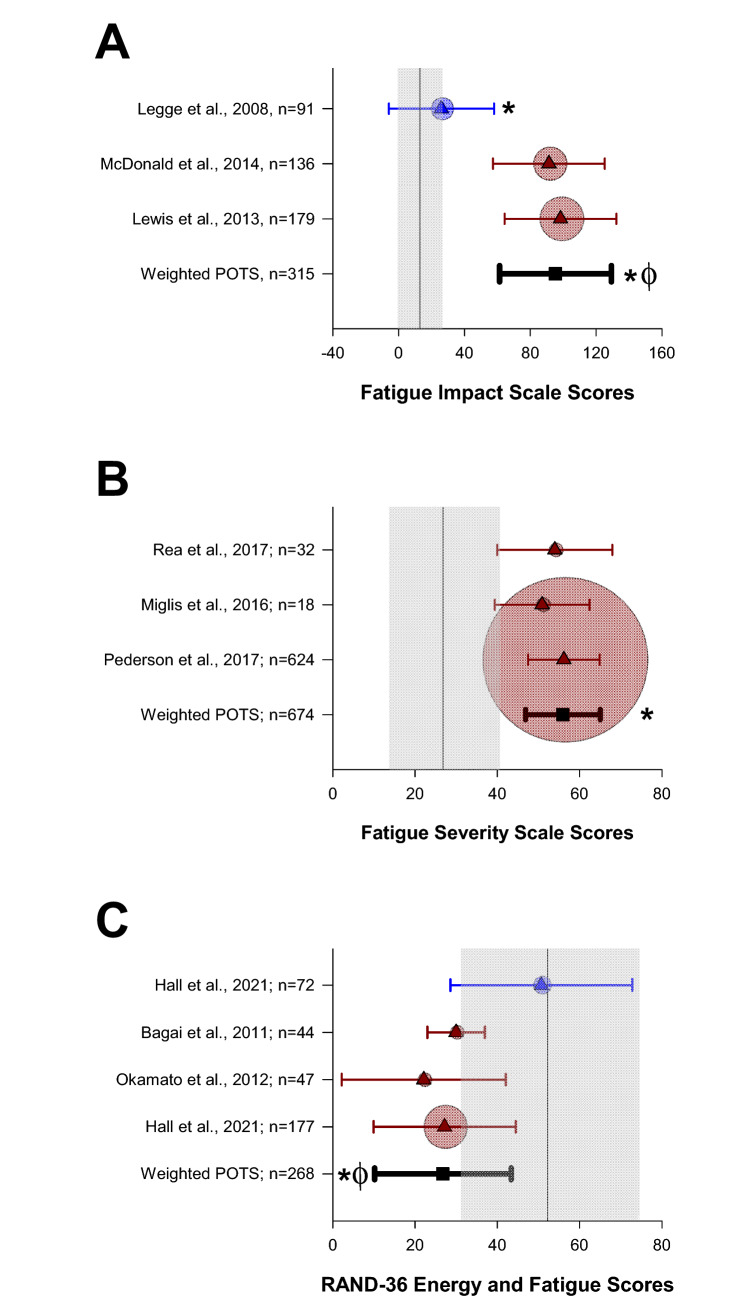
Meta-analysis of Fatigue Impact Scale (FIS) (**A**), fatigue severity scale (FSS) (**B**), and RAND-36 energy and fatigue scores (**C**) in patients with VVS and POTS compared with normative data. Mean scores from patients with POTS (red) and VVS (blue) are represented by triangles, with whiskers denoting the standard deviation. Circles indicate the relative sample size. Weighted means and pooled standard deviations for patients with POTS are denoted with black squares and whiskers. Mean scores and standard deviation of USA reference data for the FIS (*n* = 91) [83], FSS (*n* = 16) [87], and RAND-36 (*n* = 2329) [89] are represented by the vertical line and shading, respectively. ^*^Significant difference from reference data (*p* < 0.05). ^ϕ^Significant difference from VVS (*p* < 0.05)

In Fig. 2, data from our meta-analysis can be seen, comparing available data on fatigue between patients with different forms of orthostatic syncope in comparison with reference control data.

Compared with reference data, patients with POTS and VVS had higher scores on the FIS, indicating more severe fatigue (*p* < 0.05). Based on FIS scores, patients with POTS presented with significantly more severe fatigue than patients with VVS (*p* < 0.05).

The three studies that used the FSS score to examine fatigue in patients with POTS all reported significantly higher scores, and therefore more severe fatigue, than the reference scores (*p* < 0.05).

Relative to reference data, RAND-36 scores in the energy and fatigue subdomain were significantly lower in patients with POTS compared with reference data (*p* < 0.05), but there were no significant differences in RAND-36 energy and fatigue subdomain scores between patients with VVS and the reference data (*p* = 0.567). Scores for the energy and fatigue subdomain of the RAND-36 were significantly lower (*p* < 0.05) in patients with POTS than those with VVS, indicating more fatigue and lower energy levels in those with POTS.

## Discussion

We have provided a comprehensive review of the association between fatigue and disorders of orthostatic syncope. We identified that fatigue is prevalent in patients with orthostatic syncope, with scores indicating more severe fatigue in patients with orthostatic syncope than healthy controls. We also showed that fatigue severity was dependent on the specific subtype of syncope, whereby patients with POTS were especially affected by fatigue in all domains, including both physical and mental fatigue domains. In two studies, the potential association between fatigue and CFS was also investigated, with many POTS patients also meeting diagnostic criteria for CFS, and higher fatigue scores in those with POTS who also met CFS criteria. This association between POTS and CFS may contribute to the greater fatigue severity observed in patients with POTS relative to patients with other disorders of orthostatic syncope. The results of our meta-analysis confirmed the presence of fatigue in patients with orthostatic syncope, and again highlighted the severe fatigue associated with POTS. In addition, where studies considered different domains of fatigue in patients with POTS, fatigue was determined to be multi-dimensional, with each sub-domain impacting fatigue severity equally across all domains. Finally, patients with POTS who had more severe fatigue were more likely to experience poor mental health, brain fog and reduced sleep quality.

The specific cause of fatigue in patients with orthostatic syncope is unknown, but is suspected to result from global cerebral hypoperfusion when upright secondary to a failure to adequately compensate for the haemodynamic changes associated with the upright posture [47–50]. This is supported by data showing that, compared with healthy controls, a global reduction in cerebral blood flow and oxygenation is observed in those with chronic fatigue [51–54]. Similarly, improvements in cerebral blood flow in patients with fatigue are associated with a reduction in fatigue symptoms [55] and improvement in neurocognitive function [56]. In addition, cognitive impairments in patients with OH are position-sensitive, with decrements in cognitive function in the upright position relative to supine, further highlighting the role of orthostatic haemodynamic impairments in cognitive function, and perhaps explaining the mental fatigue reported by patients with orthostatic syncope [57]. None of the included studies provided information on the posture in which the fatigue assessments were completed, but the presumption would be that these survey-based instruments would mostly have been completed whilst sitting. It is possible that, had they been conducted standing, fatigue would have been even more profound, and that the true burden of fatigue on these patients during activities of daily living is underestimated.

We were not able to consider whether there was a temporal relationship between the onset of orthostatic syncope symptoms and fatigue, which might help elucidate causality or infer the mechanism of the association. This partly reflects that our primary question related to whether patients with orthostatic syncope experienced fatigue (rather than the causal nature of any relationship), and partly that data on the timing of onset of fatigue relative to the timing of onset of first syncopal episode were not provided in any study identified through our search criteria. However, fatigue symptoms were noted to vary with the severity of orthostatic cardiovascular dysfunction, and this might imply causality—this is supported by the observation that fatigue was more severe in those with more severe autonomic dysfunction and those with more severe orthostatic symptoms. Further study is needed to identify the nature and potential mechanisms of the association between orthostatic syncope and fatigue.

When comparing patients with either POTS or VVS, patients with POTS reported more severe fatigue and lower health-related quality of life than those with VVS [41]. In every study but one, patients with POTS were found to have more severe fatigue than healthy controls. The only study that did not find more severe fatigue in patients with POTS evaluated fatigue 1 year after diagnosis, when many patients had improved and no longer met criteria for POTS [36]. Of note, those in this study who had more severe orthostatic symptoms at the 1-year follow-up had more severe fatigue [36]. The impact of fatigue in patients with VVS was less clear, with more severe fatigue compared with controls when evaluated using the FIS [46], but not when considered using the RAND-36 fatigue subscale [41]. One further study that was not identified by our search also reported RAND-36 fatigue subscale data in patients with VVS [58], with responses (50 ± 22) remarkably similar to those (51 ± 22) reported by Hall et al. [41]. Of note, there is some overlap between the two samples that might explain the similar results reported in these two studies. The lack of significant fatigue in the two studies by Hall et al. and Ng et al. may reflect that the patients with VVS included in these studies were younger, and had no comorbid conditions [41, 58]—it may be that fatigue is more concerning in older adults with VVS and/or with comorbid conditions [46]. This is supported in part by the observation that older age was associated with more severe fatigue in adults with OH [40]. Patients with POTS are more likely to be diagnosed with other comorbidities than patients with VVS [59, 60], and the presence of these other comorbid conditions may also contribute to the difference in fatigue severity between the two patient populations. CFS, in particular, has been frequently observed in those with POTS [61, 62] and could exacerbate the cognitive impairments and fatigue associated with POTS (the association between POTS and fatigue is so prevalent that the designation “CFS-POTS” has been coined to reflect those who are diagnosed with POTS but also present with fatigue that meets the criteria for CFS). As might be expected, those with CFS-POTS were more fatigued than those with non-CFS-POTS, reflecting the association between the two conditions [38]. This raises the question of whether there is diagnostic, mechanistic or treatment overlap between these two conditions [63–65]. Indeed, some have suggested that POTS could be a subset of CFS, with the two conditions sharing a similar underlying mechanism [66]. However, one key feature of symptoms of CFS is that they are not relieved by sleep (and therefore a supine position) [67], and this is distinct from patients with POTS, in whom symptoms of fatigue are typically reduced when supine, presumably reflecting the mechanistic link between fatigue and orthostatic reductions in cerebral blood flow in patients with POTS [68]. It may be that evaluation of fatigue in the supine position would be beneficial in distinguishing between POTS and CFS. Certainly, patients with POTS had more severe fatigue than healthy controls, regardless of the presence of a CFS diagnosis, highlighting the need to consider fatigue, and its management, in patients with POTS. Finally, whether the presence of fatigue in patients with POTS is equally associated with all subtypes of POTS (hyperadrenergic, hypovolemic or neuropathic) is not known and should be considered in future studies.

More studies were identified examining fatigue in patients with POTS, and this may reflect the high prevalence of fatigue in this disorder. However, the comparative lack of data on fatigue in patients with other orthostatic disorders represents a concerning knowledge gap and does not necessarily mean that fatigue is not prevalent in these disorders but rather that it is not well studied. For example, only one study examined the association between OH and fatigue, observing more severe fatigue in patients with OH than a control cohort, particularly in those with the delayed subtype of OH [40]. Given this initial finding, and the known association between impaired orthostatic cardiovascular control and cognitive function [57, 69], this study highlights the need for greater consideration of fatigue in patients with OH. Similarly, few studies reported on the association between fatigue and VVS, with disparate results, also highlighting the need for greater focus on fatigue in patients with VVS.

The mental health of patients with POTS with fatigue was also adversely affected. Patients with POTS who were fatigued were more likely to die by suicide and had a higher frequency of suicide attempts than healthy controls [42]. This is somewhat surprising as patients with POTS have frequently been noted to have similar lifetime prevalence for major depressive disorders and anxiety compared with healthy controls [70, 71]. However, whilst major depressive disorders were prevalent at similar levels to healthy controls, mild depressive symptoms were common in patients with POTS, perhaps as a consequence of living with a chronic illness [70, 72]. The association between fatigue and mental health is not clear, but it has been suggested that it reflects that many people with POTS suffer from sleep disturbance, pain, fatigue and brain fog, which can severely diminish quality of life and lead to suicidal ideation [42]. Psychiatric comorbidities are also noted in patients with VVS, with a higher prevalence than healthy controls [8, 73]. Whether this also reflects an association between fatigue and mental health concerns in patients with other orthostatic syncope disorders is unclear. However, in the general population, fatigue is consistently associated with mental health concerns and depression [74]. Therefore, considering psychological conditions when treating individuals with POTS and VVS may be of benefit, and psychological interventions could also reduce the negative impacts on mental health associated with fatigue [75].

Brain fog was noted to be another important symptom associated with fatigue and POTS. It has been proposed that brain fog is a cognitive complaint similar to mental fatigue, and this was reflected in the more impaired mental fatigue subdomain scores in patients with POTS [35, 76]. Brain fog has been described to impede cognitive performance in patients with POTS, and the cause of brain fog has been attributed to a reduction in cerebral blood flow [77]. Accordingly, cerebral hypoperfusion may not only be linked to fatigue but also to the cognitive deficits seen in patients with POTS, with the cognitive decline observed being attributed to excessive levels of synaptic norepinephrine [78]. The strong associations between fatigue, poor sleep quality and brain fog support emerging evidence that addressing fatigue and sleep concerns, ideally supplemented by exercise training (if tolerated), may improve brain fog, as well as physical symptoms, in patients with POTS [79]. Of note, serotonin-norepinephrine reuptake inhibitors worsen cognitive symptoms in patients with POTS, supporting the notion that high levels of synaptic noradrenaline play a role in the symptoms [78]. While brain fog is less commonly reported in other orthostatic syncope disorders, there are signs that impaired orthostatic cardiovascular control and coincident reductions in cerebral blood flow are associated with impaired cognitive function in children and adults [69], and in children, the severity of orthostatic intolerance is noted to predict classroom effort and have important implications for their schooling [80].

Intriguingly, general fatigue severity was not related to disease severity in patients with POTS, inferred from the magnitude of the heart rate increment observed during head up tilt. This is in keeping with previous observations that the orthostatic heart rate response is also not a good predictor of quality of life in patients with POTS [8, 75]. Healthcare providers should not overlook the possibility for severe fatigue in patients with POTS, even those with less severe orthostatic tachycardia.

Patient and public involvement in this project was represented by Syncope Trust and Reflex Anoxic Seizures (STARS), a non-profit organization that unites individuals, families and medical professionals to provide support, education and promote research for patients with syncopal disorders. Our results resonated with our stakeholder communities, patient partners and patient advocacy groups, who noted that, for patients with orthostatic syncope, fatigue is relentless, debilitating and exhausting, with a negative impact on self-esteem and quality of life. They felt that symptoms of fatigue were often dismissed by healthcare professionals, or misdiagnosed, adding to the stress and anxiety of living with these conditions [81]. For some, particularly those with POTS, the associated fatigue is so severe and unremitting that it limits their ability to be engaged in full-time employment and complete activities of daily living [37, 81]. These observations are entirely in keeping with the findings of this systematic review and meta-analysis, and further highlight the importance of considering fatigue when evaluating and treating patients with orthostatic syncope in order to improve their quality of life.

One limitation of the present analysis is that, for the most part, it was not possible to evaluate the impact of age, sex or race on fatigue severity in patients with orthostatic syncope, because these data were rarely provided. With regards to the effects of sex on fatigue, one study reported that male patients with POTS were more fatigued than females [41]. Given the female predominance in patients with POTS [59], further investigation with a larger cohort of males is needed to further elucidate the relationships between sex and fatigue in patients with orthostatic syncope. Whether there are sex differences in fatigue in patients with other orthostatic syncope disorders is not known. Another limitation of the present analysis is that no studies were identified that considered the associations between RAS in children and fatigue, or CSH and fatigue in older adults, and there were limited data in patients with VVS and OH. We were also limited in general by the small number of studies identified, and this may reflect that this is an area that is understudied in the field. It is possible that, despite using broad search criteria in a number of academic databases, some relevant studies were not identified by our search, largely reflecting that indexing standards for fatigue instruments and a lack of standardized reporting may have influenced our ability to identify relevant studies. We did not evaluate the quality of evidence for the studies identified because it is not relevant to our research methodology. For our research question, study design is not likely to have influenced the quality of the extracted data; for example, baseline data from the placebo arm of a randomized controlled trial would not be of higher quality than those from a cross-sectional study. Similarly, these quality-of-evidence assessments are usually performed for the primary outcome of the study, which would not be pertinent to data on fatigue because they were often a secondary outcome measure. Heterogeneity analyses were also not performed because they are not valid in meta-analyses with only a small number of studies to compare, and can only be attempted in analyses like these where the outcome measures are the same, which limits their utility and interpretation. Heterogeneity analyses are also not particularly useful in this case because in fact the overarching theme in the analyses was remarkably homogeneous—fatigue was consistently identified as a concern in patients with orthostatic syncope. As described above, only two studies reported that fatigue was not more severe in patients with orthostatic syncope, and both were in younger populations with no comorbidities. We transparently reported the raw data, effect sizes, statistical comparisons and any provided variables that might have influenced study results to enable comparison of different studies. Finally, we considered the possibility for bias in the selection of the articles included. All decision-making criteria were objective and non-contentious, adhering to the inclusion and exclusion criteria outlined, and conducted in duplicate by two independent reviewers, and thus are unlikely to have influenced article selection or the results of the meta-analysis.

## Conclusion

Fatigue is a common symptom that is prevalent in patients with orthostatic syncope. Fatigue was noted to be problematic in patients with POTS, older patients with VVS, and patients with OH, and was particularly severe in patients with POTS. Fatigue is associated with negative effects on sleep quality, social/physical function, cognitive function, brain fog and mental health. Despite the evidence that fatigue is prevalent in patients with orthostatic syncope, with negative impacts on quality of life, it remains poorly studied in this population. Researchers and clinicians should prioritize consideration of the associations between orthostatic syncope and fatigue.

## Supplementary Information

Below is the link to the electronic supplementary material.

Supplementary file1 (DOCX 36 KB)
