# Correction to: Faintly tired: a systematic review of fatigue in patients with orthostatic syncope

**DOI:** 10.1007/s10286-022-00886-x

**Published:** 2022-09-20

**Authors:** Ryan E. Y. Wu, Farhaan M. Khan, Brooke C. D. Hockin, Trudie C. A. Lobban, Shubhayan Sanatani, Victoria E. Claydon

**Affiliations:** 1 Department of Biomedical Physiology and Kinesiology, Simon Fraser University, Burnaby, BC Canada; 2 Syncope Trust and Reflex Anoxic Seizures Group (STARS) and Arrhythmia Alliance, Stratford-upon-Avon, Warwickshire, UK; 3 Department of Pediatrics, University of British Columbia, Vancouver, BC Canada

## Correction to: Clinical Autonomic Research (2022) 32:185–203 10.1007/s10286-022-00868-z

The formatting of Tables 1 and 3 have been incorrectly published in original publication.

The Supplementary Tables 1 and 2 have also been incorrectly published in original publication.

The original article has been corrected now.

