# Supplementary material for: Faintly tired: a systematic review of fatigue in patients with orthostatic syncope
Source: Clin Auton Res. 2022 Jun 10;32(3):185–203. doi: 10.1007/s10286-022-00868-z (PMC9186485; doi:10.1007/s10286-022-00868-z)
Supplement: Supplementary file 1 — Supplementary file1 (DOCX 36 KB) [file 10286_2022_868_MOESM1_ESM.docx]

**Supplementary Table 1: Search Strategy**

| **Database** | **Search Criteria** | **# of results** |
| --- | --- | --- |
| **MEDLINE**  **(PubMed)** | (syncope OR presyncope OR faint* OR "vasovagal syncope" OR POTS OR "postural orthostatic tachycardia syndrome" OR "orthostatic hypotension" OR "autonomic failure" OR "carotid sinus hypersensitivity") OR "orthostatic intolerance" OR "loss of consciousness" OR "drop attack" OR syncope[MeSH Terms] OR presyncope[MeSH Terms] OR fainting[MeSH Terms] OR syncope, vasovagal[MeSH Terms] OR postural orthostatic tachycardia syndrome[MeSH Terms] OR pure autonomic failure[MeSH Terms] OR orthostatic intolerance[MeSH Terms] OR orthostatic hypotension[MeSH Terms]) OR loss of consciousness[MeSH Terms] OR drop attack[MeSH Terms])  AND (fatigue OR fatigue[MeSH Terms] OR "brain fog") | 1,190 |
| **Web of Science** | (syncope OR presyncope OR faint* OR "vasovagal syncope" OR POTS OR "postural orthostatic tachycardia syndrome" OR "orthostatic hypotension" OR "autonomic failure" OR "carotid sinus hypersensitivity" OR "orthostatic intolerance" OR "loss of consciousness" OR "drop attack")  AND (fatigue or "brain fog") | 1,134 |
| **PsychINFO** | As for Web of Science | 125 |
| **CINAHL** | As for Web of Science | 348 |
| **Total** |  | 2,797 |

**Supplementary Table 2: Summary of Fatigue Instruments**

| **(i) Instrument**  **(ii) Instrument type**  **(iii) Frequency (# of studies)** | **Assessment Details** | **Scoring** | **Reference Data**  **(Mean ± Standard Deviation)** |
| --- | --- | --- | --- |
| (i) Chalder Fatigue Scale  (ii) Fatigue  (iii) 1 | This scale measures self-reported fatigue. It was originally developed as a 14-item scale, but was reduced to 11-items based on enhanced discriminative properties with the removal of 3 items that were not specific to fatigue. The scale contains two subdomains that measure mental (4 items) and physical (7 items) fatigue. | This instrument has two scoring systems. In the identified study the bimodal scoring system was used, where respondents answer each question with a 1 or a 0 to indicate whether the questions apply to them or not (subscale range 0-7; total score range 0-14). Higher scores indicate greater fatigue. | Reference data not applicable because the data were not scored according to the standard approach. |
| (i) Checklist of Individual Strength (CIS)  (ii) Fatigue  (iii) 1 | This 20-item questionnaire was designed to measure several aspects of fatigue. The checklist has four subscales: subjective fatigue (8 items), motivation (4 items), physical activity (3 items), and concentration (5 items). | Each item is scored on a 7-point Likert scale (range 1-7). Higher scores correspond with increased fatigue and concentration problems, and with lowered motivation and physical activity. Subscale scores are the sum of the item scores. A fatigue subscale score >36 represents severe fatigue (range 8-56). | Netherlands Reference Data (n=1923) [94]  Fatigue: 23.0±10.8  Concentration Impairment: 12.4±6.0  Motivation: 11.1±4.7  Physical Activity: 8.3±4.3  Total: 54.8±21.5 |
| (i) Fatigue Impact Scale (FIS)  (ii) Fatigue  (iii) 3 | The FIS is a 40-item symptom-specific measure that evaluates the effect of fatigue on three domains of daily life: cognitive functioning; physical functioning and psychosocial functioning. . | Patients rate each item on a scale of 0 to 4, with 0 representing no problem and 4 representing an extreme problem. The sum of the 40 individual items is used to generate the total score (range 0-160). Higher total scores indicate more severe fatigue. | USA Reference Data (n=91) [89]:  13 ± 14 |
| (i) Fatigue Severity Scale (FSS)  (ii) Fatigue  (iii) 3 | The FSS is a nine question self-report measure of fatigue severity from the past week. The FSS is an indicator of the impact of fatigue on functional ability. | Each question from the scale is scored on a scale from 1-7 points. The total score for the scale ranges from 9 to 63, with higher scores indicating increased fatigue. Scores >36 indicate excessive fatigue. | USA Reference Data (n=16) [93]:  26.8 ± 13.4 |
| (i) Fatigue Visual Analogue Scale (F-VAS)  (ii) Fatigue  (iii) 1 | This scale is a single-item scale which measures the severity of fatigue with a specific question, “How much of a problem has fatigue or tiredness been for you in the past week.” | This scale scores the single item on a 0 to 10 scale, with higher scores indicating more fatigue experienced. | USA Reference Data (n=46) [88]:  2.8 ± 2.5 |
| (i) Multidimensional Fatigue Inventory (MFI)  (ii) Fatigue  (iii) 1 | This measure is a self-report instrument used to assess fatigue as experienced by patients. The MFI contains 20 items organized into 5 sub-scales: general fatigue, physical fatigue, reduced activity, reduced motivation, and mental fatigue. | Each item within the instrument is scored between 1 to 5, with higher scores indicating more fatigue. Each subscale has a maximum score of 20. | USA Reference Data (n=222) [90]:  General Fatigue: 12.9 ± 4.7  Physical Fatigue: 10.9 ± 4.4  Reduced Activity: 9.3 ± 4.2  Reduced Motivation: 9.6 ± 3.9  Mental Fatigue: 10.9 ± 4.5 |
| (i) Myalgic Encephalomyelitis/chronic Fatigue Syndrome  Fatigue Type Questionnaire (MFTQ)  (ii) Fatigue  (iii) 1 | The MFTQ consists of 22 questions related to the severity, frequency, and onset of fatigue within five proposed dimensions of fatigue: post-exertional, wired, brain fog, energy, and flu-like fatigue This measure is used to assess fatigue variations in patients with chronic fatigue syndrome. | Fatigue severity is scored on a 0 to 100 scale where a higher score indicates higher symptom severity for the participant. The frequency for items is then scored on a five-item Likert-scale: with 1=never, 2=seldom, 3=often, 4=usually, and 5=always. Individual item scores are created by multiplying the frequency score by the severity score. | USA Normative Data (n=251) [91]:  Post-Exertional: 10.69 ± 25.44  Wired: 32.7 ± 50.2  Brain Fog: 25.8 ± 48.4  Energy: 20.9 ± 42.7  Flu-Like: 34.9 ± 48.9  Total: 127.6 ± 179.2 |
| (i) Orthostatic Hypotension Symptoms Assessment (OHSA)  (ii) Fatigue  (iii) 1 | The OHSA is comprised of six questions, which ask the participants to rate the level of intensity of their symptoms, including fatigue | This assessment uses an 11-point scale, where 0 = no symptom and 10 = the worst possible symptom. Patients are asked to assess the severity of these symptoms over the past week. | USA Reference Data (n=184) [40]:  Fatigue sub scale 2.2 ± 2.6 |
| (i) RAND 36-Item Health Survey  (ii) Health Related Quality of Life (QoL)  (iii) 3 | This scale is composed of 36 items which assesses eight health-related domains: physical functioning, role limitations due to physical health, role limitations due to emotional health, social functioning, emotional well-being, energy and fatigue, pain, and general health. Low scores in the energy and fatigue domain indicate more severe fatigue. | The scores in each domain of the scale range from 0 to 100, with higher scores representing greater levels of functioning and a preferable health-related quality of life. | USA Normative Data (n=2329) [95]:  Energy and fatigue: 52.2 ± 22.4 |
| (i) Wood Mental Fatigue Inventory (WMFI)  (ii) Fatigue  (iii) 2 | This scale is comprised of nine questions that relate to how troublesome specific mental fatigue symptoms have been in the last month. | Fatigue is scored on a five-item Likert-scale that ranges from 0=not at all, 1=a little, 2=somewhat, 3=a lot, and 4=very much. Higher scores indicate more severe symptoms. | UK Reference Data (n=144) [92]:  7.7 ± 5.1 |
